# Choline Supplementation Sensitizes *Legionella dumoffii* to *Galleria mellonella* Apolipophorin III

**DOI:** 10.3390/ijms21165818

**Published:** 2020-08-13

**Authors:** Marta Palusińska-Szysz, Agnieszka Zdybicka-Barabas, Rafał Luchowski, Emilia Reszczyńska, Justyna Śmiałek, Paweł Mak, Wiesław I. Gruszecki, Małgorzata Cytryńska

**Affiliations:** 1 Department of Genetics and Microbiology, Institute of Biological Sciences, Faculty of Biology and Biotechnology, Maria Curie-Sklodowska University, Akademicka St. 19, 20-033 Lublin, Poland; 2 Department of Immunobiology, Institute of Biological Sciences, Faculty of Biology and Biotechnology, Maria Curie-Sklodowska University, Akademicka St. 19, 20-033 Lublin, Poland; barabas@poczta.umcs.lublin.pl (A.Z.-B.); cytryna@poczta.umcs.lublin.pl (M.C.); 3 Department of Biophysics, Institute of Physics, Faculty of Mathematics, Physics and Computer Science, Maria Curie-Sklodowska University, Maria Curie-Sklodowska Square 1, 20-031 Lublin, Poland; rafal.luchowski@poczta.umcs.lublin.pl (R.L.); wieslaw.gruszecki@poczta.umcs.lublin.pl (W.I.G.); 4 Department of Plant Physiology and Biophysics, Institute of Biological Sciences, Faculty of Biology and Biotechnology, Maria Curie-Sklodowska University, Akademicka St. 19, 20-033 Lublin, Poland; e.reszczynska@poczta.umcs.lublin.pl; 5 Department of Analytical Biochemistry, Faculty of Biochemistry, Biophysics and Biotechnology, Jagiellonian University, Gronostajowa 7 St., 30-387 Krakow, Poland; justyna.smialek@doctoral.uj.edu.pl (J.Ś.); pawel.mak@uj.edu.pl (P.M.)

**Keywords:** *Legionella*, apolipoprotein, phosphatidylcholine, atomic force microscopy, fluorescence lifetime imaging microscopy

## Abstract

The growth of *Legionella dumoffii* can be inhibited by *Galleria mellonella* apolipophorin III (apoLp-III) which is an insect homologue of human apolipoprotein E., and choline-cultured *L. dumoffii* cells are considerably more susceptible to apoLp-III than bacteria grown without choline supplementation. In the present study, the interactions of apoLp-III with intact *L. dumoffii* cells cultured without and with exogenous choline were analyzed to explain the basis of this difference. Fluorescently labeled apoLp-III (FITC-apoLp-III) bound more efficiently to choline-grown *L. dumoffii*, as revealed by laser scanning confocal microscopy. The cell envelope of these bacteria was penetrated more deeply by FITC-apoLp-III, as demonstrated by fluorescence lifetime imaging microscopy analyses. The increased susceptibility of the choline-cultured *L. dumoffii* to apoLp-III was also accompanied by alterations in the cell surface topography and nanomechanical properties. A detailed analysis of the interaction of apoLp-III with components of the *L. dumoffii* cells was carried out using both purified lipopolysaccharide (LPS) and liposomes composed of *L. dumoffii* phospholipids and LPS. A single micelle of *L. dumoffii* LPS was formed from 12 to 29 monomeric LPS molecules and one *L. dumoffii* LPS micelle bound two molecules of apoLp-III. ApoLp-III exhibited the strongest interactions with liposomes with incorporated LPS formed of phospholipids isolated from bacteria cultured on exogenous choline. These results indicated that the differences in the phospholipid content in the cell membrane, especially PC, and LPS affected the interactions of apoLp-III with bacterial cells and suggested that these differences contributed to the increased susceptibility of the choline-cultured *L. dumoffii* to *G. mellonella* apoLp-III.

## 1. Introduction

Legionellosis is an important but uncommon bacterial respiratory infection with varying severity, i.e., from the flu-like infection called Pontiac fever which does not require specialized treatment, to acute multilobar pneumonia called Legionnaires’ disease which can result in death. Humans can be infected in both the living environment (community-acquired pneumonia, CAP) and during a hospital stay (nosocomial pneumonia). The infection may have a sporadic or outbreak form [1,2]. The diseases are caused by bacteria belonging to the *Legionellaceae* family, which are ubiquitous in water and soil environments. *Legionella* species can survive in all elements of systems supplying water from the natural sources to the tap. *Legionella* spp. contaminates water sources such as air conditioning, cooling towers, whirlpool spas, as well as medical and industrial equipment function simultaneously as amplifiers or disseminators of these bacteria. The bacteria can be inhaled into the lungs, where they invade alveolar macrophages. The capability of *Legionella* spp. to intracellularly proliferate in immune cells (designed to kill bacteria), and therefore use them as host cells, is a prerequisite for development of the disease. In Europe, 5500 to 6500 cases of Legionnaires’ disease are reported annually [3]. In the USA, the rate of reported legionellosis cases increased from 0.42 to 1.62 per 100,000 persons in 2000–2014 [4]. The high percentage of therapeutic failures (the mortality rate 11% to 33% in intensive care units) could be related to the intracellular location of the bacteria in human monocytes and macrophages, and therefore the low efficiency of antibiotics that are most commonly used in pneumonia treatment [5].

To date, 65 species of bacteria from the *Legionellaceae* family have been isolated from both natural and clinical sources (http://www.bacterio.cict.fr/l/legionella.html). In the USA and Europe, the most common cause of legionellosis was *L. pneumophila*, which has been responsible for approximately 90% of culture-confirmed cases. Serogroup 1 was predominant (84.2%), and serogroups 2–3 (7.4%) accounted for the remaining cases. However, based on isolation from patients, *L. dumoffii* has been regarded as one of 28 non-*L. pneumophila* species that have been reported to be pathogenic in humans [6,7]. Among non-*L. pneumophila* species, *L. dumoffii* causes about 10% of Legionnaires’ disease cases, which are often fatal, especially in immunocompromised patients [8]. Pneumonia which is caused by this microorganism is more serious and progresses more rapidly in humans than that caused by other *Legionella* spp. [9]. In addition to lung infection, *L. dumoffii* is responsible for pericarditis, prosthetic valve endocarditis, and septic arthritis [10,11,12].

An efficiently functioning bacterial envelope is essential for communication between the *Legionella* cell and its phagotrophic host. *L. dumoffii* does not produce a capsule or an exopolysaccharide. Therefore, the predominant molecule presented on the cell surface of these bacteria is the lipopolysaccharide (LPS). On the one hand, LPS functions as a non-fimbrial adhesin, which determines colonization of host cells, and thus becomes an important factor of *Legionella* virulence involved in the complex mechanisms of the disease development. On the other hand, LPS is an efficient barrier that protects bacteria from environmental factors, drugs, and hydrophobic substances [13]. LPS localized in the outer layer of the outer membrane consists of the following three regions: O-antigen, core and lipid A. The lipid A region anchors LPS molecules to the outer membrane, through hydrophobic interactions with the acyl chains of phospholipids (PLs) constituting the inner layer of this membrane [14].

Phosphatidylcholine (PC), i.e., a phospholipid that is a common membrane component of eukaryotic cells, also constitutes an important part of *Legionella* spp. cell membranes [15]. The presence of PC in a cell envelope is required for the successful interaction of *L. pneumophila* with the host cell [16]. It has been demonstrated that various *Legionella* spp. can synthesize PC from exogenous choline [17,18,19,20]. In *L. dumoffii*, structurally different PC species are localized in both the inner and outer membranes. Interestingly, *L. dumoffii* cultured in the presence of exogenous choline exhibits a reduced capability of TNF-α induction, which enables the bacteria to evade the host immune system [19].

The lipid-binding protein, apolipophorin III (apoLp-III) is an insect homologue of human apolipoprotein E (apoE) engaged in lipid transport, as well as immune response in insects. The protein is an exchangeable component of lipophorin particles. Similarly to the apoE molecule, whose N-terminal domain forms a bundle of four α-helices in an up-and-down spatial arrangement, the apoLp-III molecule is a bundle of five antiparallel α-helices, in which hydrophobic chains are directed to the interior and hydrophilic chains are exposed to the environment [21,22]. In addition to lipid binding, insect apoLp-III exhibits carbohydrate-binding properties [23,24]. These features facilitate apoLp-III interactions with different macromolecules, for example, bacterial LPS and lipoteichoic acid (LTA), and fungal β-1,3-glucan [24,25,26,27]. It has been demonstrated that apoLp-III of the greater wax moth *Galleria mellonella* bound to the cell surface of different bacteria and fungi, which was accompanied by antimicrobial action of the protein [28,29,30,31,32]. Our previous results revealed the susceptibility of *L. pneumophila* and *L. dumoffii* to antibacterial action of *G. mellonella* apoLp-III [33,34,35]. Our detailed Fourier transform infrared (FTIR) spectroscopy study indicated interactions of apoLp-III with LPS and lipid molecules of *L. pneumophila* cell envelope taking place during binding to the bacterial cell surface [35]. Interestingly, as presented in our recent paper, human apoE, similarly to insect apoLp-III, bound *L. pneumophila* LPS and caused alterations in the *L. pneumophila* cell surface [36].

Unlike *L. pneumophila*, *L. dumoffii* cells cultured in the presence of exogenous choline were considerably more susceptible, that is, they showed three times lower survival rate, after treatment with *G. mellonella* apoLp-III at a concentration of 0.4 mg/mL, than the bacteria grown without supplementation of this compound [33]. These results combined with the documented capability of utilization of exogenous choline for PC synthesis by *Legionella* [18,19] suggested that the increased PC content in the cell membrane could be responsible for the observed effects [33]. Indeed, a comparative lipidomic analysis revealed that cell membranes of *L. dumoffii* grown on choline-supplemented medium exhibited a ca. 12% increase in the content of PC in favor of phosphatidylethanolamine (PE) as compared with bacteria cultured without exogenous choline [37]. Furthermore, detailed examination carried out by means of attenuated total reflection Fourier transform infrared (ATR-FTIR) spectroscopy demonstrated considerable differences in apoLp-III binding to lipid bilayer membranes prepared from lipids extracted from *L. dumoffii* cells grown with and without choline supplementation. In addition, fluorescence lifetime imaging microscopy (FLIM) measurements revealed that apoLp-III interacted with considerably higher affinity and penetrated more deeply the liposomes formed of lipids extracted from choline-cultured *L. dumoffii*, i.e., containing a higher PC level [37].

The aim of this work was to analyze whether the presence of LPS had an impact on the documented differences in the interaction of apoLp-III with *L. dumoffii* grown with and without choline supplementation or whether these differences were mainly due to changes in the composition of the outer membrane phospholipids caused by choline supplementation. To elucidate the molecular basis of the higher susceptibility of choline-grown *L. dumoffii* to *G. mellonella* apoLp-III, apoLp-III interactions with intact *L. dumoffii* cells cultured without and with exogenous choline were assessed in the present study. The effects of apoLp-III binding to the bacterial cells on their surface topography and nanomechanical properties were analyzed by atomic force microscopy (AFM). The binding ability of apoLp-III to the bacterial cells was compared by using fluorescently labeled apoLp-III and fluorescence lifetime imaging microscopy (FLIM). The characterization of the *L. dumoffii* LPS was performed for the first time. Formation of *L. dumoffii* LPS and apoLp-III complexes was analyzed by sodium dodecyl sulfate-polyacrylamide gel electrophoresis (SDS-PAGE) and size exclusion chromatography. Furthermore, a detailed analysis of apoLp-III interactions with the phospholipids and LPS of the *L. dumoffii* cell envelope were performed on a liposomal model using FLIM. The use of PLs and LPS isolated from non- and choline-supplemented *L. dumoffii* allowed analysis of apoLp-III interactions with systems that reflected the natural layout containing various PLs species, in contrast to models based on defined components.

## 2. Results

### 2.1. Effects of Apolipophorin III on the L. dumoffii Cell Surface

**Figure 1:**
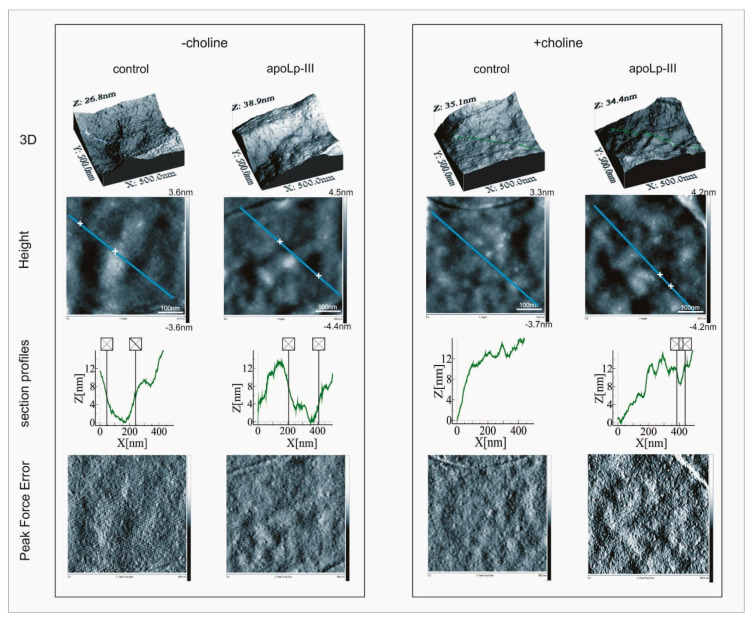
Atomic force microscopy imaging and analysis of the *L. dumoffii* cell surface. Bacteria grown without or with exogenous choline (−choline and +choline, respectively) were incubated without (control) or with *G. mellonella* apolipophorin III (apoLp-III) (0.1 mg/mL) for 1 h, and then imaged by atomic force microscopy (AFM). Representative three-dimensional (3D) images, peak force error, and height maps of 500 × 500 nm cell surface areas are presented. The section profiles correspond to the blue lines marked in the height maps.

AFM imaging revealed that, irrespective of the culture conditions and apoLp-III treatment, the *L. dumoffii* cell surface was decorated with very small, mostly regular granules, which were well visible on the bacterial height maps (Figure 1).

However, an analysis of the section profiles demonstrated the presence of recesses (ca. 8–10 nm in depth and ca. 200 nm in width) in the cell surface of the bacteria grown without the choline addition. No such recesses were detected in the cell surface of the bacteria cultured on the choline-supplemented medium (Figure 1).

Moreover, the AFM analyses demonstrated that the culture conditions affected the cell surface properties of *L. dumoffii* (Table 1).

The bacteria grown without or with choline supplementation (−choline, +choline, respectively) were incubated without or in the presence of apoLp-III (−apoLp-III, +apoLp-III, respectively) at the concentration 0.1 mg/mL, and then analyzed by AFM. The results are presented as ±SD. The differences between two mean values were established using the U Mann–Whitney’s test.

Growth in the presence of exogenous choline resulted in a ca. two-fold increase in the Derjaguin–Muller–Toporov (DMT) modulus, reflecting elasticity, which indicated a change in the nanomechanical properties of the cell envelope. Furthermore, considerable alterations in the cell surface properties resulted from the apoLp-III treatment of *L. dumoffii* cells. The cell envelope elasticity was affected most severely by apoLp-III. ApoLp-III caused a ca. six-fold increase in the cell surface elasticity in the bacteria grown without choline in contrast to the choline-cultured *L. dumoffii* whose elasticity did not change after the incubation with apoLp-III. Although the cell surface roughness and adhesion forces seemed not to be affected by the culture conditions, they both were influenced by the apoLp-III treatment (Table 1).

**Figure 2:**
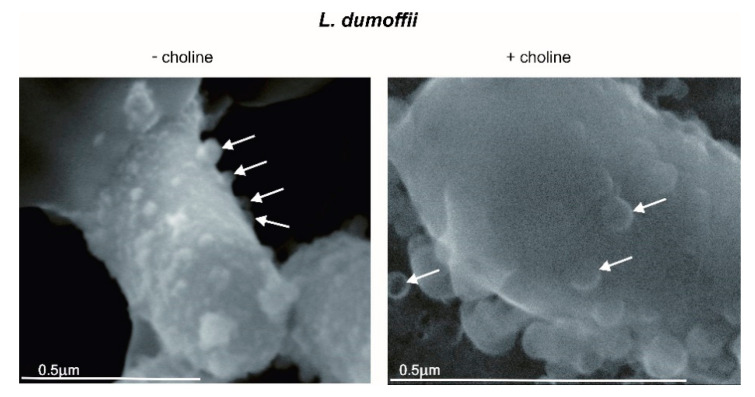
SEM images of *L. dumoffii* cells grown on the medium with and without choline with visible outer membrane vesicles (OMVs) (arrows).

The scanning electron images of *L. dumoffii* cells cultured on the medium with and without choline showed that the bacteria produced vesicles, which were present on the cell surfaces (Figure 2).

To confirm that the vesicles were formed from the protuberances of the outer membrane, X-ray elemental microanalysis of the *L. dumoffii* cell surface, as well as released vesicles and those visible on the bacterial cell surface, was performed using SEM. The comparison of the obtained results revealed a very similar elemental composition, which confirmed the origin of the vesicles (Table 2).

The SEM images and X-ray analysis showed that *L. dumoffii* cultured on the medium with choline and without choline supplementation produced outer membrane vesicles (OMVs).

### 2.2. Interaction of Fluorescently Labeled Apolipophorin III with L. dumoffii Cells

The fluorescently labeled apolipophorin III (FITC-labeled apoLp-III) bound to the *L. dumoffii* cells, as demonstrated by fluorescence microscopy imaging (Supplementary Figure S1). Importantly, apoLp-III exhibited higher affinity to the bacteria grown on the choline-supplemented medium as compared with those cultured without the addition of exogenous choline. The increase in fluorescence could be a result of an increase in the number of binding sites. The difference was well reflected by the 26.9% (±11.48) higher level of light integrated density calculated for the cells exposed to apoLp-III for 15 min.

### 2.3. Fluorescence Lifetime Imaging Microscopy (FLIM) Analysis of ApoLp-III Interaction with L. dumoffii Cells

**Figure 3:**
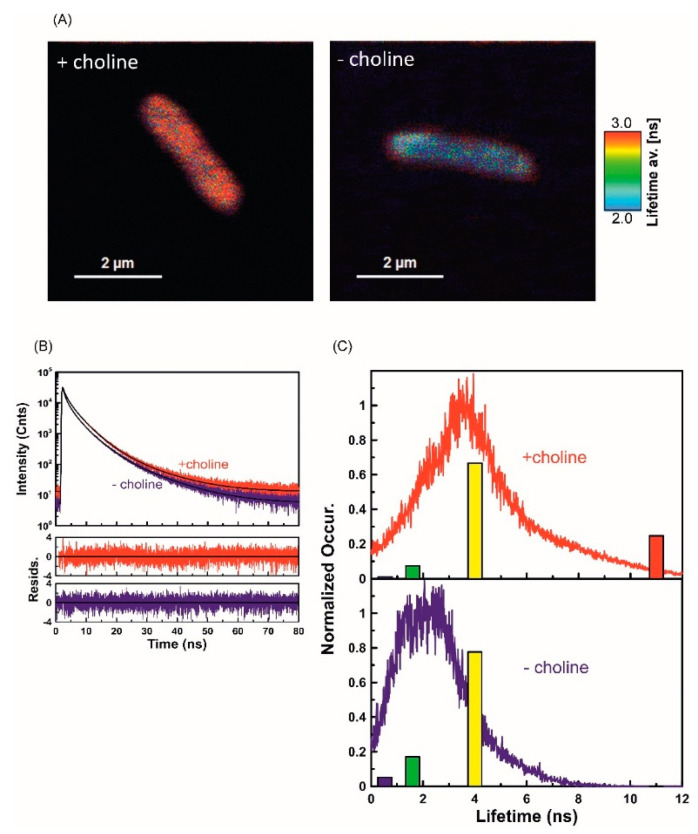
(**A**) Fluorescence lifetime images of a single *Legionella* cell incubated with FITC-labeled apoLp-III. The bacteria were grown without and with exogenous choline (−choline and +choline, respectively). The color bar placed on the right of the images is the lifetime scale from 2 ns (blue) to 3 ns (red). Images were acquired with a MicroTime 200 confocal lifetime microscope. The samples were excited with a 470 nm pulse of light and emission was collected through a 488 long-pass filter. The longer fluorescence lifetime is indicative of deeper penetration of FITC-apoLp-III into the cell membrane structure. More than 20 individual cells of each type were analyzed and the images show representative effects; (**B**) Decays of fluorescence intensity following pulse excitation recorded for a single cell of *Legionella* bacteria treated with FITC-apoLp-III. The bacteria were grown with (red) and without (blue) choline supplementation. The residuals of the best four exponential fits (black line) are shown in the bottom panels. The time of bacterial incubation (1.5 h) with apoLp-III was kept the same for both samples, 470 nm laser light was used for excitation, and the observation was performed through a 488 long wavelength pass filter. The longer fluorescence lifetime is indicative of deeper penetration of FITC-apoLp-III into the cell membrane structure; (**C**) Normalized histograms of average lifetime distribution taken from pictures presented in (A). For exponential lifetime, deconvolution was applied to build the following histograms: τ1 = 0.53 ns, τ2 = 1.6 ns, τ3 = 4 ns, τ4 = 11 ns in both panels represented by bars with a height proportional to their percentage contribution. Characteristically, bacteria grown without exogenous choline (−choline) incubated with apoLp-III did not show the presence of a relatively long (11 ns) lifetime component.

As indicated by the results of our previous study of phospholipids isolated from *L. dumoffii cells*, incorporation of FITC-apoLp-III to the lipid membrane environment increased considerably the fluorescence lifetime, i.e., from 3.2 ns in the water phase to 31.1 ns [37]. Such a pronounced effect has been attributed to penetration of the fluorophore moiety into the hydrophobic region of the membrane. In the present study, this effect was applied to study the FITC-apoLp-III binding to *L. dumoffii* cells. Figure 3A presents images of intact bacterial cells obtained with application of the FLIM technique. Two types of bacteria were studied, i.e., those cultured on the choline-supplemented medium and control bacteria grown without choline addition. As can be seen, FITC-apoLp-III interacted with both types of cells; hence, fluorescence emission clearly distinguished the bacteria against the dark background in the images. Interestingly, the fluorescence lifetimes, represented by false colors (the scale displayed in the figure), are different in the two types of cells (Figure 3A). Such an effect represents different modes of interaction of FITC-apoLp-III with bacterial membranes. In general, longer fluorescence lifetimes can be observed in the case of the cells cultured in the choline supplementation conditions. The observation is supported by the fluorescence decay analysis, based on all the photons collected during the microscopic imaging process (Figure 3B). Precise fluorescence lifetime analyses of the images presented in Figure 3A (so-called full FLIM) are displayed in Figure 3C showing histograms of the average lifetime distribution. Satisfactory analysis of the fluorescence decays recorded (exponential deconvolution), required application of the following four lifetime components: τ_1_ = 0.53 ns, τ_2_ = 1.6 ns, τ_3_ = 4 ns, and τ_4_ = 11 ns. Figure 3C also presents relative amplitudes of the fluorescence lifetime components represented by color bars with different heights proportional to amplitudes. As can be seen, in the case of *Legionella* cells cultured with choline supplementation, there is a long-lifetime component (τ4 = 11 ns) that has not been detected in the control (Figure 3C). The results indicate greater effectiveness of apoLp-III binding and deeper penetration into the cell membrane of *L. dumoffii* cultured on the choline-supplemented medium.

### 2.4. Characteristics of L. dumoffii Lipopolysaccharide (LPS)

#### 2.4.1. Electrophoretic Analysis of Lipopolysaccharide (LPS)

**Figure 4:**
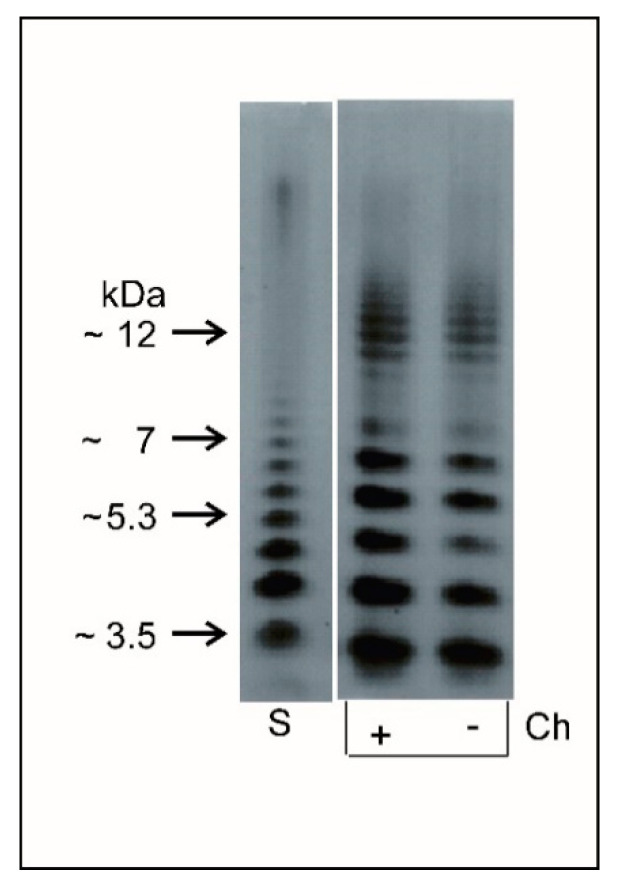
Electrophoretic analysis of *L. dumoffii* lipopolysaccharide (LPS). LPS phenol-soluble fraction of *L. dumoffii* cultured without (−Ch) and with (+Ch) choline supplementation visualized by silver staining. Lane S, *Salmonella enterica* serovar Typhimurium LPS reference.

In order to analyze the interactions of apoLp-III with *L. dumoffii* cell envelope components more comprehensively and to answer the question whether PLs or LPS play an essential role in this interaction, *L. dumoffii* LPS was purified and characterized for the first time. The *L. dumoffii* LPS was isolated by hot phenol-water extraction from delipidated and enzymatically digested cells. The SDS-PAGE profile of the phenol-phase LPS of bacteria grown with and without choline showed a characteristic S-type form LPS profile with distinct ladder-like bands covering low and high molecular mass regions (Figure 4). The LPS profile exhibited three fractions: rough LPS (R-type LPS), a fraction containing from one to five subunits with molecular mass ranging from 4 to 7 kDa, and a fraction with seven to approximately 15 subunits with molecular mass from 10 to 12 kDa.

#### 2.4.2. Fatty Acid Profile and Sugar Analyses

Fatty acids in the LPS preparations were analyzed with GLC-MS of methyl esters. The fatty acid profile including 26 both linear and methyl branched-chain (*anteiso* and *iso*) acids was dominated by amide-linked 3-hydroxy acids (Table 3). The LPS of *L. dumoffii* contained 18 3-hydroxy fatty acids in the range of C12–C21 and 8 non-hydroxylated acids (C14–C17); 3-hydroxy fatty acids were dominated by *n*3-OH 14:0 (10%), *a*3-OH 18:0 (9%), and *n*3-OH 16:0 (8%). Long chain (ω-1)-oxo fatty acid (27-oxo-octacosanoic) and (1,ω)-dioic acids (heptacosane-1,27-dioic and nonacosane-1,29-dioic) were also found in the *L. dumoffii* LPS; 27-oxo-octacosanoic acid was the major fatty acid in the LPS.

The GLC-MS sugar analyses of the alditol acetates derived after full acid hydrolysis of the LPS revealed the presence of mainly: quinovosamine, mannose, glucose, galactose, glucosamine, galactosamine, and 2,3-diamino-2,3-dideoxy-d-glucose (Table 4). Quinovosamine was the major sugar in the LPS.

The composition of fatty acids and sugars in LPS derived from the phenol and water phases exhibited quantitative but not qualitative differences. There were no differences in the fatty acid and sugar profiles between the LPSs isolated from bacteria cultured on the medium with or without choline supplementation.

### 2.5. Interaction of Apolipophorin III with L. dumoffii Lipopolysaccharide (LPS)

**Figure 5:**
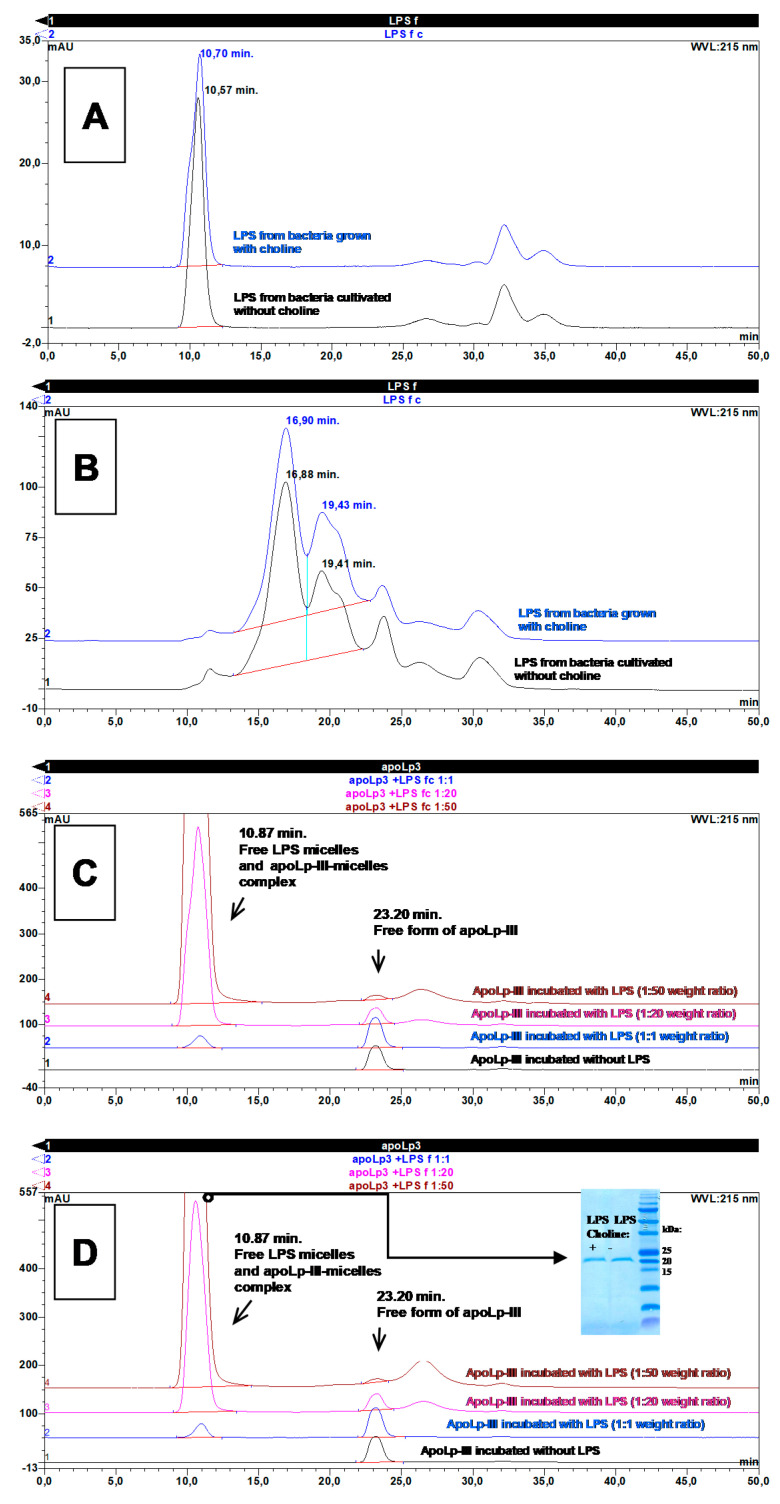
Analysis of *G. mellonella* apoLp-III interactions with *L. dumoffii* LPS by size exclusion chromatography. (**A**) Elution profiles of LPS pre-incubated in physiological conditions without the presence of apoLp-III. The retention times obtained are shorter than the retention times of Blue Dextran 2000 (11.26 min.), suggesting that the analyzed LPS preparations form micelles with molecular mass equal or larger than 2 MDa; (**B**) Elution profiles of LPS denatured in the presence of SDS. The two dominant peaks visible for each LPS preparation correspond to molecular masses of 167.3 and 73.1 kDa; (**C**) Elution profiles of apoLp-III alone and apoLp-III incubated with LPS isolated from bacteria supplemented with choline at 1:1, 1:20, and 1:50 protein to LPS mass proportions; (**D**) Elution profiles of apoLp-III alone and apoLp-III incubated with LPS isolated from bacteria grown without choline at 1:1, 1:20, and 1:50 protein to LPS mass proportions. The insert shows SDS-PAGE image of 10.87 min peaks collected from separation of the apoLp-III-LPS complex at the 1:50 mass ratio. The protein band at approximately 20 kDa was analyzed with the use of an automatic protein sequencer and identified as *Galleria mellonella* apoLp-III.

Since the ability of apoLp-III to interact with the LPS of other Gram-negative bacteria has been demonstrated [25,26,35], we were interested to determine whether apoLp-III could interact with *L. dumoffii* LPS. To understand the binding interaction in more detail, the complex formed between LPS and apoLp-III was characterized using size exclusion chromatography (SEC). During the SEC analyses in physiological saline (PBS), the *L. dumoffii* LPS obtained from both bacteria supplemented with choline and those grown without supplementation gave single peaks eluting at 10.70 and 10.57 min, respectively (Figure 5A). However, both these elution times were shorter than the retention time of Blue Dextran 2000 (11.26 min), indicating that the analyzed LPS preparations formed micelles with molecular mass were equal or higher than 2 MDa. Identical results were obtained for LPS separations carried out in the buffer containing 6 M GuaHCl on the one hand, suggesting that the micelles are stable in chaotropic agents. On the other hand, boiling of the LPS preparations in the SDS detergent effectively disrupted these micelles, as shown in the chromatograms presented in Figure 5B. Both studied LPS preparations formed relatively fuzzy peaks in SDS-containing buffer with two dominant maxima at 16.9 and 19.4 min, which corresponded to molecular masses of 167.3 and 73.1 kDa, respectively. Both these masses were in good agreement with the average masses of different monomeric LPS forms isolated from outer membranes of many species of Gram-negative bacteria [38].

The formation of the complex between apoLp-III and *L. dumoffii* LPS was verified after pre-incubation of both compounds at the following three different protein to lipid mass ratios: 1:1, 1:20, and 1:50. The percentage of protein bound to LPS was calculated taking into account the decrease in the apoLp-III peak area in relation to the peak area of protein incubated without LPS. As presented in Figure 5C (for LPS isolated from bacteria supplemented with choline) and Figure 5D (for LPS from bacteria grown without choline supplementation), the area of the apoLp-III peak (retention time 23.2 min), for the protein incubated alone and incubated with LPS at a 1:1 mass ratio, is the same.

Additionally, the incubation of apoLp-III with higher amounts of LPS causes a gradual decrease in the apoLp-III peak, suggesting that the protein forms complexes with *L. dumoffii* LPS. Assuming measured protein peak areas, the amount of protein in the free form for the LPS preparation isolated from bacteria supplemented with choline, at the 1:20 protein to LPS mass ratio, is 66.2% (in relation to the peak area of protein incubated without LPS). In turn, the percentage of the free form of protein for LPS from bacteria grown without choline supplementation is 60.6%. At the 1:50 mass ratio of protein and LPS from choline-supplemented bacteria, 18.2% of apoLp-III occurs in the free form, while the percentage of the free form of protein for LPS isolated from *L. dumoffii* grown without choline supplementation accounts for 11.8%. To confirm the formation of a complex between apoLp-III and LPS micelles at the 1:50 mass ratio, the fraction containing the LPS micelle peak eluting at 10.87 min (Figure 5C,D) was collected and analyzed by SDS-PAGE (insert in Figure 5D). The band of protein at approximately 20 kDa was analyzed using an automatic protein sequencer, yielding the DASTPLQDLE N-terminal amino acid sequence, typical for *G. mellonella* apolipophorin III. This result unequivocally proves that this protein formed stable complexes with *L. dumoffii* LPS micelles in our experiments, as in the case of LPS isolated from *E. coli* reported by Oztug et al. [26]. Taking into account all the results from the abovementioned experiments, it can be assumed that a single micelle of *L. dumoffii* LPS is formed from 12 to 29 monomeric LPS molecules and that one *L. dumoffii* LPS micelle binds two molecules of apoLp-III (the apoLp-III peak disappears completely at approximately 50-fold mass excess of LPS, therefore, one can assume that single micelle of 2 MDa molecular weight binds 40 kDa of apoLp-III, what is equivalent to ca. two molecules of apoLp-III).

### 2.6. FLIM Analysis of Apolipophorin III Interaction with Liposomes

**Figure 6:**
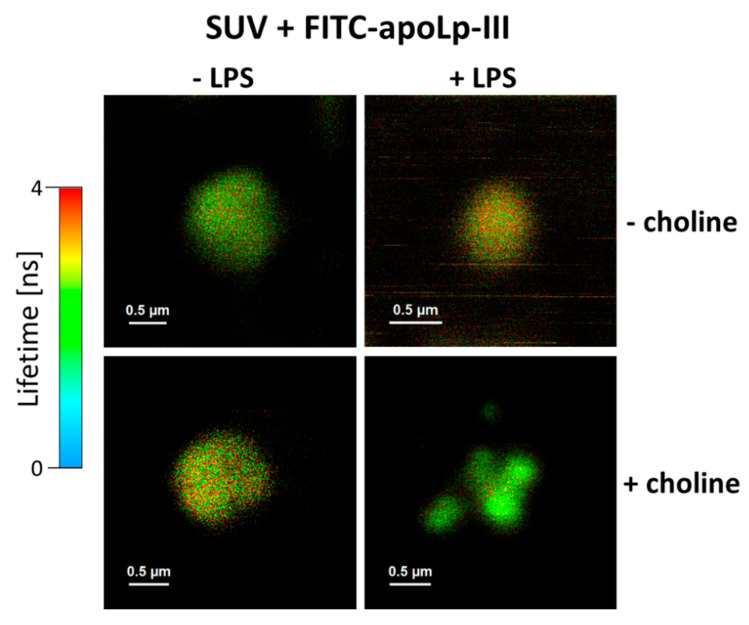
Fluorescence lifetime imaging microscopy (FLIM) images of small unilamellar vesicles (SUVs) exposed to FITC-apoLp-III (Apo). Liposome membranes were formed with phospholipids of *L. dumoffii* cells cultured in the presence (+Chol) and absence (−Chol) of exogenous choline with (+LPS) or without (−LPS) *L. dumoffii* LPS. Note the liposome decomposition in the sample (+LPS, +Chol).

A liposome model was applied to examine the interaction of apoLp-III with lipid membranes revealed the following: Small unilamellar vesicles (SUV) formed with the lipid components of *L. dumoffii* cells cultured in the presence (+Chol) and absence (−Chol) of exogenous choline and giant unilamellar vesicles (GUV) formed with DPPC. Lipids isolated from bacteria cultured on the medium with choline contained more PC (12%) and less phosphatidylethanolamine (approximately the same range) as compared with lipids from bacteria cultured on the medium without choline supplementation [37]. To resolve the role of LPS in the apoLp-III interaction with the *L. dumoffii* cell envelope, purified *L. dumoffii* LPS was used for formation of SUVs, in addition to *L. dumoffii* PLs. ApoLp-III was labeled with FITC which enabled examination of binding of the protein to lipid membranes by means of fluorescence lifetime imaging microscopy (FLIM). Figure 6 shows the FLIM images of SUV membranes formed with (+) or without (−) purified *L. dumoffii* LPS, recorded after exposure to apoLp-III. As can be seen, the interaction of the protein with membranes formed of lipids extracted from cells cultured in the presence of exogenous choline and containing LPS as an additional component of liposomes, resulted in apparent degradation of lipid membranes, manifested by the liposome fragmentation. 

**Figure 7:**
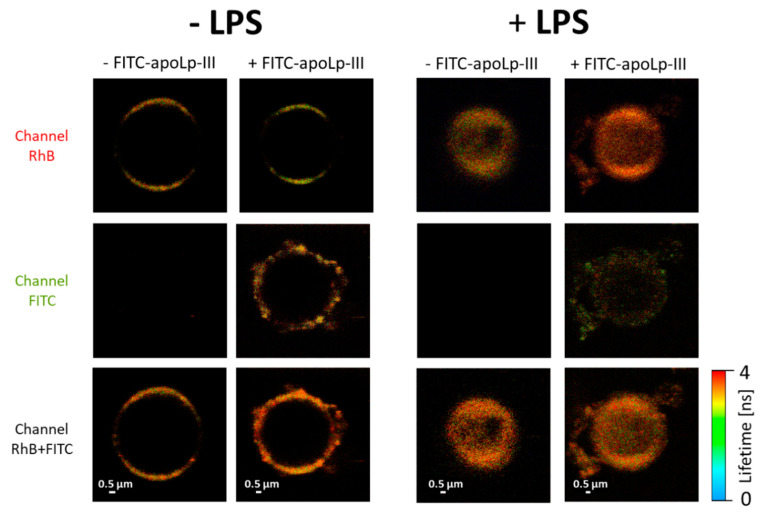
FLIM images of giant unilamellar vesicles (GUVs) formed with DPPC with (+LPS) or without (−LPS) *L. dumoffii* LPS. The vesicles were exposed (+apoLp-III) or not exposed (−apoLp-III) to FITC-apoLp-III. The upper panel shows the fluorescence emission recorded in the channel representing Rhodamine B (RhB) present in the lipid phase and the middle panel shows fluorescence emission recorded in the channel representing FITC (Figure S2 for a specification of the fluorescence emission channels). The lower panel shows superposition of fluorescence signals recorded in both channels.

Such an effect of a destruction of lipid membranes was observed in all the replicates of this experiment. In order to examine the mechanisms associated with the interaction of apoLp-III with lipid membranes in detail, we applied a GUV model (Figure 7). Unilamellar lipid vesicles were formed with DPPC labeled with a fluorescence probe rhodamine B (RhB). The fact that the fluorescence emission photons of RhB and FITC, excited by the same 470 nm laser, could be detected in separate spectral channels (Supplementary Figure S2), facilitated parallel recording of images of the lipid phase of the membranes and interacting protein molecules, respectively. As can be seen from the images presented in Figure 7, apoLp-III binding to liposomes results in a pronounced modification of the membrane surface, manifested by the presence of bulk nanostructures. The RhB channel (Figure 7, upper panels) shows a well-ordered lipid phase, manifested by orientation of fluorophores in the membrane plane (low fluorescence intensity in the lefthand and righthand sections of the liposome [39]), both in the samples with and without apoLp-III. This means that the irregular structures formed in the surface region of the lipid membranes exposed to apoLp-III (FITC channel) are predominantly composed of the protein. Such an effect is also clearly pronounced in membranes with incorporated *L. dumoffii* LPS (Figure 7, righthand panels). Interestingly, the LPS-modified liposomes show a greatly heterogeneous surface as compared to the membranes formed with pure DPPC. Exposure of the LPS-containing vesicles to apoLp-III results in formation of extramembraneous structures visible in both the lipid and protein channels. In our opinion, this is a demonstration of the severe effect of the structural properties of membranes and disruption of the membrane integrity. The combined effect of LPS and apoLp-III, reported in Figure 7, was observed in all the examined GUV samples.

## 3. Discussion

In the present study, the interactions of *G. mellonella* apoLp-III with intact *L. dumoffii* cells cultured without and with exogenous choline were analyzed and compared. Fluorescently labeled apoLp-III bound more efficiently to *L. dumoffii* grown on the choline-supplemented medium. Furthermore, the cell envelope of these bacteria was penetrated more deeply by FITC-apoLp-III, as demonstrated by detailed FLIM measurements. The results obtained using the whole bacterial cells confirmed the results of our previous study on apoLp-III interactions with lipid bilayer membranes. *G. mellonella* apoLp-III bound with higher affinity and penetrated the bilayer membranes and liposomes formed of lipids extracted from choline-supplemented *L. dumoffii* more deeply than those composed of lipids isolated from bacteria cultured without choline [37].

Previous studies have demonstrated the susceptibility of *L. pneumophila* and *L. dumoffii* to *G. mellonella* apoLp-III. Interestingly, in contrast to *L. pneumophila*, *L. dumoffii* cultured on the choline-supplemented medium after treatment with apoLp-III showed a three times lower survival rate than the bacteria grown without choline supplementation [33,35]. We also reported on utilization of exogenous choline by *L. dumoffii* for synthesis of PC that was subsequently built into the outer and inner cell membrane [19]. A detailed lipidomic analysis revealed increased content of PC (and an accompanying decrease in PE) in cell membranes of *L. dumoffii* grown in the presence of choline as compared with non-supplemented bacteria [37]. These results indicated that the differences in the content of phospholipids affected the interaction of apoLp-III with the examined membranes and intact bacterial cells and suggested that these differences could be a cause of the increased susceptibility of the choline-cultured *L. dumoffii* to *G. mellonella* apoLp-III. Interestingly, a study conducted by Zhang et al. [40] on apoLp-III interaction with different zwitterionic, anionic, and nonionic lipids demonstrated weaker interactions of apoLp-III with PE as compared with PC, supporting the particularly important role of PC in apoLp-III interactions with bacterial membranes; both classes of phospholipids exhibited different properties, i.e., PC formed a bilayer structure, whereas PE made the membrane more rigid [41]. Probably, a lower content of PE could result in less tightly packed membranes, resulting in membrane defects providing opportunities for apoLp-III to bind. We also provided evidence that the increased susceptibility of the choline-cultured *L. dumoffii* to *G. mellonella* apoLp-III was associated with the changed composition of membrane phospholipids, which altered the cell surface properties (e.g., elasticity) and, as a consequence, affected the interactions with apoLp-III. Interestingly, Verdon et al. [42] reported on changes in *L. pneumophila* tolerance to the antimicrobial peptide warnericin RK, resulting from alterations in the composition and length of fatty acids in phospholipid molecules. 

In order to resolve the role of LPS in apoLp-III interactions with *L. dumoffii*, LPS purified from these bacteria was used in the present study. We demonstrated that apoLp-III can form stable complexes with *L. dumoffii* LPS. Furthermore, the presence of *L. dumoffii* LPS in GUVs formed from DPPC considerably increased the interaction of apoLp-III with these liposomes. Interestingly, the incorporation of the LPS into SUVs composed of PLs from the choline-supplemented *L. dumoffii* resulted in fragmentation of the liposomes upon the interaction with apoLp-III. This indicates that LPS is an important component of the *L. dumoffii* cell envelope which facilitates apoLp-III binding; however, it is the differences in the PC content that are mainly responsible for stronger interaction of apoLp-III with the membrane, and consequently, for the higher susceptibility of choline-cultured bacteria to *G. mellonella* apoLp-III.

It is known that PC plays an important role in the interaction between *L. pneumophila* and its eukaryotic host. *L. pneumophila* mutants deficient in PC formation are less virulent than the respective wild type in their interaction with the host [16]. Our results clearly indicate an essential role of PC and changes in the lipid membrane composition in the interaction of *G. mellonella* apoLp-III with *L. dumoffii* cells. The increased content of PC in the cell membrane promoted apoLp-III binding to bacterial cells which, in consequence, increased the susceptibility of *L. dumoffii* to apoLp-III. It can be hypothesized that PC, which is important for interactions with the host and for establishing a replicative niche in its cell, also plays a role of “a receptor” for potential antibacterial molecules, for example, apoLp-III. However, binding of such molecules to PC could mask the presence of this phospholipid on the bacterial surface and impede the interaction of the bacteria with the host cell. Consequentially, this would prevent the establishment of a replicative niche and intracellular development of the bacteria. Moreover, since the choline-cultured *L. dumoffii* with increased PC content in the cell membrane exhibited reduced capability of TNF-α induction [19], such PC masking could prevent evasion of the host immune system by the bacteria by maintaining a proper level of induction of this cytokine.

Similar to other Gram-negative bacteria, *L. pneumophila* produces and releases OMVs from the cell envelope. Extramembrane vesicles, containing virulence-associated proteins, consist of a double lipid membrane, which, similar to the outer membrane of bacteria, is composed of LPS localized in the outer leaflet and PLs in the inner leaflet [43,44]. The microvesicles play a significant role in the pathogenesis and dissemination of the infection in the organism via interactions with host cells [45]. In addition to the well-known role of OMVs in bacterial pathogenesis involving transport of virulence factors to host cells, these nanovesicles take part in defence mechanisms against external threats, including antibiotics and antimicrobial peptides [46]. In the present study, we demonstrated for the first time that *L. dumoffii* cultured on the medium with and without exogenous choline can produce OMVs. Our study of the apoLp-III interaction with the components of the *L. dumoffii* cell envelope was performed on a model of liposomes (SUVs) formed of PLs with incorporated LPS isolated from the bacteria. Such liposomes mimic *L. dumoffii* OMVs. Therefore, our results suggest that this type of vesicles can be destroyed upon apoLp-III treatment. This could be considered to be a possible way to fight against *Legionella* and provides a new interesting idea for the development of alternative anti-*Legionella* therapies.

## 4. Materials and Methods

### 4.1. Bacterial Strain and Culture Conditions

*Legionella dumoffii* ATCC 33279 (American Type Culture Collection, Manassas, VA, USA) was cultured for 3 days at 37 °C on buffered charcoal yeast agar (BCYE) medium (Thermo Fisher Scientific, Waltham, MA, USA) or on a medium enriched with 100 µg/mL choline chloride (Sigma-Aldrich, St. Louis, MO, USA). Bacteria cultured on the BCYE medium with and without exogenous choline were harvested with 0.5 M NaCl and centrifuged at 8000× *g* for 20 min. The cell pellets were washed once with 0.5 M NaCl and once with distilled water and lyophilized. Approximately 2 g of bacterial mass were obtained from *L. dumoffii* cultured with and without choline and subjected to isolation of phospholipids (PLs) and lipopolysaccharide (LPS). 

### 4.2. Preparation of L. dumoffii Phospholipids

PLs were isolated from the bacterial mass according to the Bligh and Dyer method, as previously described [37]. In this method, the organic layer contained PLs together with legioliulin which is a compound of *L. dumoffii* responsible for the blue and white autofluorescence under long-wavelength UV light [47]. Phospholipids were separated from legioliulin by one-dimensional thin-layer chromatography (TLC) on silica gel 60 F254 plates (Sigma-Aldrich, St. Louis, MO, USA, 20 × 20 cm). The plate was washed twice with chloroform:methanol (1:1, v/v) and activated at 180 °C before use. The organic fraction containing PLs and legioliulin (about 1 mg) was applied about 1 cm from the bottom of the silica gel as a narrow band. The chromatogram was developed with chloroform/methanol/acetic acid (98:2:1, v/v/v). PLs were visualized with iodine vapor, and legioliulin was detected under long-wavelength UV (Transiluminator UV-953, Sigma-Aldrich, St. Louis, MO, USA). Subsequently, the PL-containing band was scraped off, transferred to screw-capped tubes, and extracted from silica gel with a mixture of chloroform/methanol (1:2, v/v).

### 4.3. Isolation, Purification, and Electrophoretic Analysis of L. dumoffii Lipopolysaccharide (LPS)

Delipidated and dried cell pellets were suspended in 50 mM phosphate buffer (pH 7.0) supplemented with 5 mM EDTA and digested with lysozyme (6 mg/g, 16 h, 4 °C). Nucleic acids were digested by RNAse and DNAse (0.3 mg/g, 37 °C, 2 h), and then polypeptides were hydrolyzed with proteinase K (20 h, 1 mg/g, followed by incubation for 10 min at 60 °C). LPS was extracted from digested cells three times with aq 45% phenol at 68 °C [48]. The LPS containing phenol and water phases were dialyzed extensively against tap and distilled water, purified by ultracentrifugation (105,000× *g*, 4 °C, 4 h), and freeze-dried. The LPS species distributed between the phenol and water phases as hydrophobic and hydrophilic fractions accounted for, respectively, approximately 2% and 1% of the dry mass of bacteria cultured on both the choline-supplemented and non-supplemented medium. The LPSs were further analyzed to determine their fatty acid and sugar composition. LPS from the phenol phase, due to its higher yield, was used for formation of liposomes and investigation of interactions with apoLp-III.

The LPSs isolated from bacterial masses harvested from the medium with or without choline were analyzed by sodium dodecyl sulfate-polyacrylamide gel electrophoresis (SDS-PAGE). Briefly, 4.8% and 12% polyacrylamide-bisacrylamide gels were used as the stacking and separating gels, respectively. LPS (2 µg) was mixed with a sample buffer (0.5 M Tris-HCl, pH 6.8, 5% SDS, 35% glycerol, 0.1% bromophenol blue) in a 1:1 ratio (v/v). LPS bands were visualized using the silver staining method [49]. LPS from *Salmonella enterica* serovar Typhimurium (Sigma-Aldrich, St. Louis, MO, USA) was used as a standard.

### 4.4. Compositional Analyses of L. dumoffii Lipopolysaccharide (LPS)

#### 4.4.1. Fatty Acid Analysis

For fatty acid analysis, an LPS sample (1 mg) was subjected to hydrolysis in 4 M HCl (100 °C, 4 h). Free fatty acids were extracted with chloroform, and then converted into methyl esters by methanolysis (0.5 M HCl in methanol, 80 °C, 1 h). Then, the solution was cooled to room temperature and the solvent was evaporated. Fatty acid methyl esters recovered by extraction with chloroform/water (1:2, v/v) were trimethylsilylated with HMDS/TMCS/pyridine (3:1:9, v/v/v) (Sigma-Aldrich, St. Louis, MO, USA) and analyzed using gas liquid chromatography and mass spectrometry (GLC-MS).

#### 4.4.2. Sugar Analysis

For neutral and amino sugar analysis, the LPS samples were hydrolyzed with 2 M trifluoroacetic acid (TFA) (120 °C, 2 h), N-acetylated, reduced with NaBD4, and acetylated with a 1:1 (v/v) pyridine-acetic anhydride mixture (100 °C, 30 min). The products were identified as alditol acetates by GLC-MS.

#### 4.4.3. Gas Liquid Chromatography and Mass Spectrometry (GLC-MS)

The methyl esters of fatty acids and the alditol acetates of monosaccharides were analyzed by GLC-MS using a gas chromatograph (Agilent Technologies, instrument 7890A, Santa Clara, CA, USA) connected to an MSD 5975C (inert XL EI/Cl) detector. The chromatograph was equipped with an HP-5ms (SLB-5ms) capillary column (30 m × 0.25 mm; Sigma-Aldrich, St. Louis, MO, USA). A temperature gradient of 150 °C (5 min) to 310 °C, at 5 °C/min, was applied, and the final temperature was kept for 10 min.

### 4.5. Apolipophorin III Purification and Fluorescent Labeling

Apolipophorin III was purified from hemolymph of *G. mellonella* (Lepidoptera: Pyralidae), as described in detail in our previous study [37]. In brief, a lipid-deprived freeze-dried methanolic extract of hemolymph was dissolved in 0.1% TFA and subjected to high-pressure liquid chromatography (HPLC) on a Discovery Bio Wide Pore C18 4.6 × 250 mm column (Sigma-Aldrich, St. Louis, MO, USA). The separation was performed using two buffer sets, i.e., A, 0.1% TFA (v/v) and B, 0.07% TFA, 80% acetonitrile (v/v), and a linear gradient from 30 to 70% of buffer B over 35 min (flow rate 1 mL/min). The fraction containing apoLp-III was freeze-dried and stored at −80 °C. The identity and homogeneity of apoLp-III was confirmed by N-terminal sequencing using an automatic protein sequencer (Procise 491, Applied Biosystems, Foster, CA, USA). The concentration of total protein in the *G. mellonella* hemolymph and methanolic extracts was determined according to Bradford [50].

The procedure of apoLp-III labeling with fluorescein isothiocyanate (FITC, isomer 1; Sigma-Aldrich, St. Louis, MO, USA) was described in detail in our previous paper [37]. Briefly, a mixture composed of apoLp-III dissolved in sodium borate pH 9.0 (2 mg of protein in 200 µL) and an equimolar amount of FITC dimethylsulfoxide solution was incubated, for 1 h, at room temperature in darkness. Then, unreacted FITC was quenched by addition of a 1 M glycine water solution (50 µL) followed by incubation of the mixture for 0.5 h in darkness. Afterwards, a sample acidified to pH 2.0 by addition of TFA was subjected to HPLC chromatography on a C18 column (identical as that mentioned above). A linear gradient from 55% to 70% of buffer B over 20 min and a 1 mL/min flow rate was applied. The fraction containing monosubstituted FITC-apoLp-III was collected and freeze-dried. Before use, the FITC-apoLp-III was dissolved in sterile water.

### 4.6. Interaction of Apolipophorin III with L. dumoffii Lipopolysaccharide (LPS)

The formation of the complex between *L. dumoffii* LPS and apoLp-III was verified by size exclusion chromatography (SEC) using a Superdex 200 10/300 GL column (GE Life Sciences, Marlborough, MA, USA) and an UltiMate 3000 HPLC system (Thermo Fisher Scientific, Waltham, MA, USA). The separations were carried out at room temperature (RT) in 50 mM sodium phosphate pH 7.4 containing 150 mM NaCl (PBS buffer), in PBS buffer supplemented with 1% (w/v) (SDS) or in PBS buffer containing 6 M guanidinum hydrochloride (GuaHCl). The flow rate was 0.7 mL/min, the spectrophotometric detection was carried out at 215 and 280 nm, and the column was calibrated using the following molecular mass standards (all from Sigma-Aldrich, St. Louis, MO, USA): Blue Dextran 2000 (2 MDa), thyroglobulin (669 kDa), ferritin (443 kDa), catalase (232 kDa), aldolase (157 kDa), conalbumin (75 kDa), ovoalbumin (43 kDa), RNAse (13.7 kDa), and tryptophan (204 Da). Estimated total and void volumes of the column and retention volumes of standard proteins were used for calculation of distribution coefficients (KAV) and plotted versus the logarithm from the molecular masses of standard proteins. Linear regression of the obtained curve was used for calculation of molecular masses of compounds resolved during chromatography. All apoLp-III and LPS mixtures, as well as their individual solutions, were pre-incubated in PBS buffer, for 1 h, at 37 °C, immediately before injection on the column in order to facilitate formation of the protein-LPS complex. Additionally, before separations, the samples analyzed in denaturing conditions (PBS buffer containing 1% SDS) were mixed with the SDS stock solution to 3% (w/v) SDS final concentration and boiled for 5 min. In turn, the samples separated in the chaotropic buffer (PBS buffer with 6 M GuaHCl) were mixed with solid GuaHCl to a final concentration of 6 M and preincubated for 15 min at RT. Fractions eluting from the column and containing the LPS-apoLp-III complex were manually collected, concentrated on a 3 kDa cut-off centrifugal ultrafilter (Pall), resolved in reducing conditions using SDS-PAGE [51], and electrotransferred on a 0.22 μm PVDF membrane (Millipore, Burlington, MA, USA). The membrane was stained using Coomassie Brillant Blue (Sigma-Aldrich, USA). The protein bands obtained were cut out and identified by determination of their N-terminal amino acid sequences by means of a PPSQ31A automatic protein sequencer (Shimadzu, Kioto, Japan).

### 4.7. Liposome Preparation, Apolipophorin III Interaction with Liposomes, and FLIM Measurements of Liposomes

#### 4.7.1. Chemicals

L-α-dipalmitoylphosphatidylcholine (DPPC) and 1,2-dipalmitoyl-sn-glycero-3-phosphoethanolamine-N-(lissamine rhodamineB sulfonyl) (ammonium salt) were purchased from Sigma-Aldrich (St. Louis, MO, USA) and Avanti Polar Lipids, Inc. (Alabaster, AL, USA), respectively.

#### 4.7.2. Giant Unilamellar Vesicles

Giant Unilamellar Vesicles (GUVs) were formed from DPPC with 1 mol% of rhodamine B-labeled lipid. Constituents of the lipid phase of liposomes were deposited onto two platinum electrodes (35 × 4 × 0.5 mm) by evaporation from the solution, under a stream of gaseous nitrogen. Residuals of organic solvents were removed during incubation for 2–3 h under vacuum. Platinum electrodes with deposited lipid films were fixed in a Teflon holder at a distance of 3 mm. The electrodes were placed in a cuvette containing a buffer solution (1.4 mL 20 mM Tricine, 10 mM KCl, pH 7.4). Electric connections were attached to an AC field supply (DF 1641A). GUV electroformation was carried out over 2 h with an applied AC sinusoidal field with 10 Hz frequency and voltage of 9 V (peak-to-peak). The temperature during the electroformation was stabilized at 45 °C (above the main phase transition of membranes formed with DPPC, ~41 °C). GUVs containing LPS were formed as described above with addition of 2 mol% of purified *L. dumoffii* LPS.

#### 4.7.3. Small Unilamellar Vesicles

Lipids (2 mg/mL) extracted from *L. dumoffii* cells cultured without and with choline supplementation were used for formation of small unilamellar vesicles (SUV). Briefly, chloroform and methanol solutions of lipids (containing 2% of *L. dumoffii* LPS, if applicable), were mixed to attain a homogeneous solution. The solvents were evaporated under a stream of nitrogen and the lipids with LPS or lipids without LPS film of the test tube were dried under vacuum for 2–3 h. A MQ water solution (usually 0.5 mL) was added to the dried film at about 35 °C and vortexed vigorously for 5 min. Next, the lipids were sonicated in ice for 3 sec with 60% amplitude and titanium probe using a VCX-130 sonicator (Sonics Inc., Newtown, CT, USA), and then heated to 35 °C. The sonication-heating cycles were repeated 3 times. Liposomes were prepared twice and FLIM imaging of at least 5 selected liposomes was performed from each series.

#### 4.7.4. Microscopy Experiments

The liposome suspensions (20 µL) were deposited on a microscope slide. Then, 2 µL of FITC-labeled apoLp-III (0.0074 µg of protein) were added and the liposomes were immediately imaged and analyzed with FLIM spectroscopy.

The experiments were performed using a two-channel MicroTime 200 (Picoquant, Berlin, Germany) confocal system coupled to an Olympus IX71 inverted microscope. The instrument was equipped with a piezo-scanner objective working in an 80 × 80 μm maximum imaging range at a nominal positioning accuracy of 1 nm. The sample was illuminated with a 470 nm pulsed laser focused on the objects of interest by a water immersion objective (Olympus Plan Apo NA = 1.2, 60×). A 75 μm diameter confocal pinhole was used. In the case of the GUV studies, the fluorescence beam was split by 50% reflecting mirror and observed simultaneously by two analyzers (Single Photon Avalanche Diodes) in separate spectral windows. One of them detected the fluorescence of FITC (by a 520/35 dichroic band-pass filter) and the other analyzer detected rhodamine (by a 580/70 band-pass filter). Both filters were provided by Chroma-AHF AnalysenTechnik. The SUV samples were studied using a single detector in a 520/35 spectral window. The microscopy system recorded fluorescence intensity and lifetime simultaneously. The intensity decays were analyzed by fitting an exponential model using SymPhoTime 64 v. 2.4 software (PicoQuant, Berlin, Germany).

### 4.8. Scanning Electron Microscopy Imaging and X-ray Analysis of Bacterial Cells

Bacteria cultured on the BCYE medium with and without choline supplementation were suspended in water to achieve optical density OD_600_ = 0.2. The suspension was mounted on an aluminum plate and left to dry at room temperature. The samples were imaged by scanning electron microscopy (SEM).

The elemental analysis of the surface of bacterial cells and outer membrane vesicles (OMV) was performed using a FEI Quanta 3D FEG scanning electron microscope (Analytical Laboratory, Faculty of Chemistry, UMCS, Lublin, Poland). Energy dispersive X-ray (EDX) microanalysis was performed using a silicon drift detector (SDD) operating at 30 kV acceleration voltage. The microanalysis was carried out without any shadowing and the concentrations of the elements were determined using the standardless method.

### 4.9. Apolipophorin III Binding to L. dumoffii Cells and Imaging of Bacteria

#### 4.9.1. Laser Scanning Confocal Microscopy

For fluorescence imaging, *L. dumoffii* cells (50 μL of suspension, OD_600_ = 0.2) grown without and with exogenous choline supplementation were incubated with FITC-apoLp-III (final concentration 0.2 mg/mL) at 37 °C, for 5 or 15 min. Then, the bacterial suspensions were centrifuged (4000× *g*, 10 min, 4 °C) and washed three times with 20 mM phosphate buffer, pH 7.4, containing 0.9% NaCl. Finally, the bacteria were suspended in 20 mM phosphate buffer pH 7.4 (10 µL) and bound to polylysine coated microscopic slides for imaging [35]. The bacteria were imaged using a laser scanning confocal microscope LSM 5 PASCAL (Carl Zeiss, Jena, Germany) (excitation and emission wavelength 470 and 520 nm, respectively, excitation time 600 ms). To compare the binding ability of FITC-apoLp-III to *L. dumoffii* grown on the choline-supplemented and non-supplemented medium, integrated density was calculated from 40 images obtained from two independent experiments using ImageJ software ver. 1.440 (rsb.info.nih.gov/ij/). Integrated density is defined as the sum of the light of pixels in the image and reflects the light intensity of the bacterial cells.

#### 4.9.2. Fluorescence Lifetime Imaging Microscopy

For fluorescence lifetime imaging microscopy (FLIM), 100 µL of a water suspension containing *L. dumoffii* cells cultured without or with exogenous choline (OD_600_ = 0.01) were incubated with FITC-apoLp-III (final concentration 0.67 µg/mL), for 1.5 h, at 37 °C. After incubation, the bacterial suspensions were spun down (4000× *g*, 10 min, 4 °C), the supernatant was removed, and the cells were gently washed once with 200 µL of sterile 10 mM phosphate buffer pH 7.4 and twice with 200 µL of non-pyrogenic water, and finally centrifuged as described above. Afterwards, the bacteria were suspended in 80 µL of non-pyrogenic water. For FLIM measurements, 10 µL of the resulting suspension was applied on the surface of a cover slip coated with polylysine and carefully rinsed three times with water after 30 sec. The FLIM measurements were carried out using a confocal MicroTime 200 system (PicoQuant, GmbH, Berlin, Germany) coupled to an OLYMPUS IX71 microscope. The samples were excited with a pulsed laser at 470 nm (repetition rate 5 MHz and time resolution 16 ps) and the photons were collected with a 60× water immersed objective (NA 1.2, OLYMPUS UPlanAPO). The restricted effective confocal volume was 0.26 fL. Scattered light was removed by a ZT 473RDC XT band pass interference filter (AnalysenTechnik, Mainz, Germany) and the observation was carried out with the use of 495 and 488 nm long-wavelength-pass filters. A single photon sensitive avalanche photodiode (τ-SPAD) was used for collection of fluorescence photons and processing was accomplished by the HydraHarp400 time-correlated single photon counting (TCSPC) module. SymPhoTime 64 software package (v. 1.6) was used for analysis of the data.

#### 4.9.3. Atomic Force Microscopy

For atomic force microscopy (AFM) imaging, 4 × 40 μL of water suspension containing *L. dumoffii* cells (OD_600_ = 0.2) cultured on the choline-supplemented or non-supplemented BCYE medium were incubated, for 1 h, at 37 °C, without (control) and with apoLp-III (final concentration 0.1 mg/mL). Then, the bacterial suspensions were centrifuged at 8000× *g* for 10 min, at 4 °C, and prepared on mica discs, as described in our previous paper [32]. The cell surface of *L. dumoffii* prepared on the mica discs was imaged using a NanoScope V AFM (Veeco, San Jose, CA, USA). The measurements were carried out in the “Peak Force QNM” operation mode using a TAP150A Antimony doped Si tip with a spring constant of 1.646 N·m^−1^ (Bruker, Santa Barbara, CA, USA). During each of the two independent experiments, three randomly chosen fields were imaged on each mica disc. The data obtained were analyzed with Nanoscope Analysis ver. 1.40 software (Bruker, USA). The values that define nanomechanical properties were calculated from 40 fields (340 × 340 nm) measured over the entire bacterial cell surface in 3 × 3 µm areas. The differences between two mean values were established using the U Mann–Whitney’s test. The section profiles and three-dimensional (3D) images of the cells were generated using WSxM 5.0 software (Nanotec, Madrid, Spain) [52].

## Figures and Tables

**Table 1:** The effect of the culture conditions and *G. mellonella* apoLp-III treatment on nanomechanical properties of *L. dumoffii* cell surface.

	Bacteria Culture Conditions (−/+Choline) and Treatment (−/+ ApoLp-III)			
	−Choline		+Choline	
	−apoLp-III	+apoLp-III	−apoLp-III	+apoLp-III
DMT modulus (MPa)	1186 (±396.46) ^a^	7414.5 (±2334.21) ^A,^*	2538 (±790.12) ^b^	2067 (±569.12) ^B^
Adhesion (nN)	0.949 (±0.03)	1.18 (±0.28) *	0.958 (±0.05)	1.146 (±0.10) *
RMS Roughness (nm)	2.293 (±0.77)	3.076 (±1.04) **	2.058 (±0.63)	3.172 (±1.27) **

The different letters in superscript indicate statistically significant differences between +choline (b, B) versus −choline (a, A) treatment. The values marked with stars in superscript indicate statistically significant differences between +apoLp-III versus −apoLp-III treatment. Statistical significance, * *p* ≤ 0.05 and ** *p* ≤ 0.01.

**Table 2:** Elemental analysis of *L. dumoffii* surface and OMV using SEM.

Elements	Surface of *L. dumoffii*		OMV	
	Weight %	Atom %	Weight %	Atom %
C	52.49 ± 35.85	58.06 ± 34.89	27.77 ± 6.88	33.30 ± 7.37
N	2.90 ± 14	2.96 ± 0.05	7.66 ± 2.34	7.95 ± 2.34
O	43.23 ± 34.28	8.42 ± 32.92	63.13 ± 8.11	58.01 ± 9.13
P	0.66 ± 0.12	0.29 ± 0.25	1.18 ± 0.87	0.56 ± 0.43
Cl	0.72 ± 0.28	0.27 ± 0.80	0.26 ± 0.6	0.18 ± 0.42

**Table 3:** Fatty acid composition of *L. dumoffii* LPS isolated from phenol phase.

No	Retention Time	Fatty Acid	Relative Content (%)
1	11.23	14:0	1
2	11.55	3-OH 12:0	tr
3	12.76	15:0	2
4	13.63	3-OH 13:0	tr
5	14.69	16:0	6
6	14.88	*i*3-OH 14:0	1
7	15.44	16:0	9
8	15.62	*n*3-OH 14:0	10
9	16.87	17:0	8
10	16.99	*a*3-OH 15:0	3
11	17.53	*n*3-OH 15:0	2
12	18.69	*i*3-OH 16:0	2
13	19.35	*n*3-OH 16:0	8
14	20.46	*i*3-OH 17:0	tr
15	20.62	*a*3-OH 17:0	tr
16	21.11	*n*3-OH 17:0	1
17	22.16	*i*3-OH 18:0	tr
18	22.77	*a*3-OH 18:0	9
19	23.78	*n*3-OH 19:0	0.5
20	23.95	*a*3-OH 19:0	1
21	24.38	*n*3-OH 19:0	2
22	25.92	3-OH 20:0	5
23	27.40	3-OH 21:0	tr
24	37.06	28:0 (27-oxo)	17
25	37.17	27:0-dioic	10
26	39.93	30:0-dioic	3

The number before colon refers to the number of carbon atoms; *a*, methyl branch at the anteiso carbon atom; *i*, methyl branch at the iso carbon atom; *n*, unbranched acid.

**Table 4:** Sugar composition of *L dumoffii* LPS from phenol phase.

Retention Time	Component	Relative Content (%)
18.18	Quinovosamine	56
19.27	Mannose	6
19.43	Glucose	2
19.61	Galactose	8
22.27	Glucosamine	17
22.64	Galactosamine	10
23.87	2,3-diamino 2,3-dideoxy-d-glucose	1

## Supplementary Materials

The following are available online at https://www.mdpi.com/1422-0067/21/16/5818/s1, Figure S1: Binding of FITC-labelled G. mellonella apoLp-III to L. dumoffii cells. Bacteria cultured without or with exogenous choline (−choline and +choline, respectively) were incubated with FITC-apoLp-III (0.2 mg/mL) for 5 or 15 minutes, and then imaged using a laser scanning confocal microscope. The small images showing individual cells are sections of the corresponding larger ones. Figure S2: Fluorescence emission spectra recorded from FITC and Rhodamine B (indicated) with marked spectral windows determined by the narrow band-pass filters applied for FLIM imaging. Excitation with a 470 nm laser.

## Abbreviations

AFM	atomic force microscopy
apoLp-III	apolipophorin III
CAP	community-acquired pneumonia
FITC	fluorescein isothiocyanate
FLIM	fluorescence lifetime imaging microscopy
HMDS	hexamethyldisilane
HPLC	high-pressure liquid chromatography
LPS	lipopolysaccharide
LTA	lipoteichoic acid
PC	phosphatidylcholine
PE	phosphatidylethanolamine
SEC	size exclusion chromatography
SEM	scanning electron microscopy
TCSPC	time-correlated single photon counting
TFA	trifluoroacetic acid
TMCS	trimethylchlorosilane
TNF-α	tumour necrosis factor α
