# Supplementary material for: Choline Supplementation Sensitizes Legionella dumoffii to Galleria mellonella Apolipophorin III
Source: Int J Mol Sci. 2020 Aug 13;21(16):5818. doi: 10.3390/ijms21165818 (PMC7461559; doi:10.3390/ijms21165818)
Supplement: Supplementary file 1 [file ijms-21-05818-s001.pdf]

Supplementary Materials:

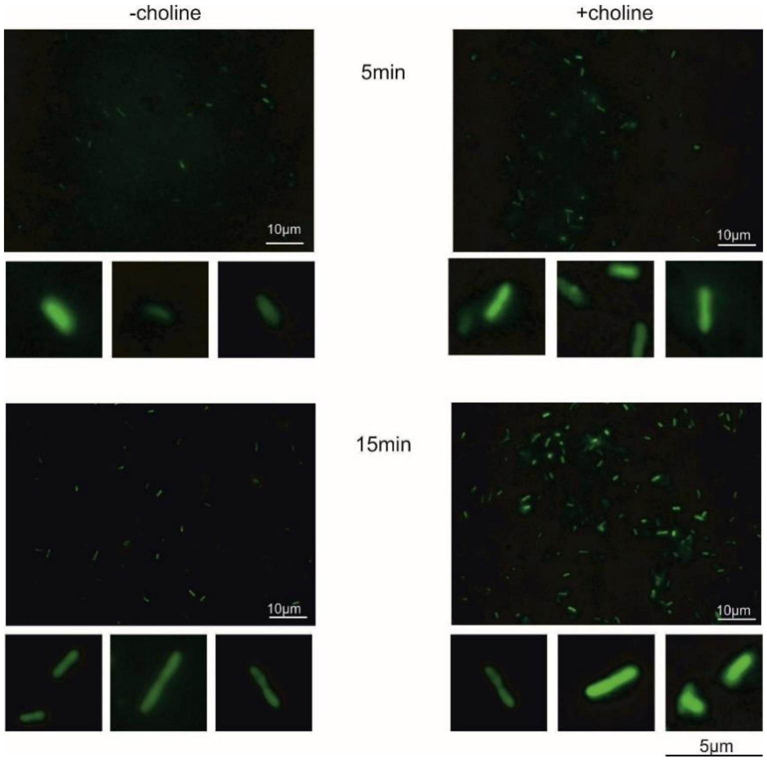

Figure S1. Binding of FITC-labelled *G. mellonella* apoLp-III to *L. dumoffii* cells. Bacteria cultured without or with exogenous choline (−choline and +choline, respectively) were incubated with FITC-apoLp-III (0.2 mg/mL) for 5 or 15 minutes, and then imaged using a laser scanning confocal microscope. The small images showing individual cells are sections of the corresponding larger ones.

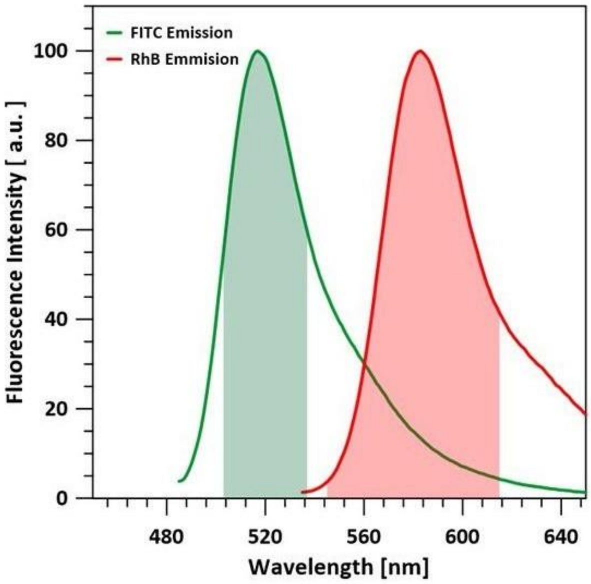

Figure S2. Fluorescence emission spectra recorded from FITC and Rhodamine B (indicated) with marked spectral windows determined by the narrow band-pass filters applied for FLIM imaging. Excitation with a 470 nm laser.
